# Effect of BPM music on post-exercise heart rate variability in male collegiate sprinters

**DOI:** 10.3389/fspor.2026.1814697

**Published:** 2026-07-20

**Authors:** Chen Bao, Jiayi Li, Ran Gao, Shixiang Zhang, Jiameng Wang

**Affiliations:** 1 Center for Art Education, Xi'an Peihua University, Xi'An, Shaanxi, China; 2 Faculty of Physical Education, Yan'an University, Yan'an city, Shaanxi, China; 3 International Nursing School of Hainan Medical University, Haikou, Hainan, China; 4 Shanghai University of Medicine and Health Sciences, Shanghai, China

**Keywords:** beats per minute, cardiac recovery, heart rate variability, high-intensity exercise, music tempo, sudden cardiac death

## Abstract

**Background:**

Sudden cardiac death (SCD) during or immediately after intense exercise remains a major concern in sports medicine. Heart rate variability (HRV) is a key indicator of autonomic regulation, and music tempo may influence post-exercise autonomic recovery. This study investigated the effects of different music tempo conditions, expressed as beats per minute (BPM), on heart rate (HR) and HRV recovery in collegiate sprinters following high-intensity exercise.

**Methods:**

Among 300 initially selected male collegiate sprinters, 265 eligible participants were included in the final analysis and randomly allocated to either a no-music control group or one of nine music intervention groups listening to music ranging from 50 to 130 BPM during recovery. Participants completed a high-intensity Wingate-style anaerobic cycling test. HR and HRV were assessed at baseline and during recovery at 0–1, 5–6, and 15–16 min after exercise. Data were analyzed using two-way repeated-measures analysis of variance with Bonferroni *post hoc* correction.

**Results:**

No significant between-group differences were observed in baseline HRV indices or anaerobic performance variables (*p* > 0.05). Significant time × group interactions were identified for HR, low-frequency (LF) power, and high-frequency (HF) power. The 120 BPM condition demonstrated comparatively favorable recovery responses, including lower HR values and beneficial autonomic modulation during recovery. However, 120 BPM was not consistently superior across all HRV parameters or recovery time points, and the 130 BPM condition showed comparable effects for several HRV indices.

**Conclusion:**

Under the present experimental conditions, music at 120 BPM was associated with relatively favorable autonomic recovery following high-intensity exercise. Nevertheless, this tempo should not be considered universally optimal. The potential role of BPM-specific music interventions in reducing SCD risk requires further investigation.

## Introduction

Athletes are exposed to a substantially greater risk of sudden cardiac death (SCD) during and immediately after high-intensity exercise than non-athletes, primarily because strenuous physical exertion can trigger underlying structural or electrophysiological cardiac abnormalities (1–3). In young athletes, the annual incidence of SCD has been estimated at 5–10 cases per million individuals (2, 4, 5). Although the overall incidence remains relatively low, ranging from 1 in 40,000 to 1 in 80,000 athlete-years, certain athletic subpopulations demonstrate markedly elevated risk (5–7). Male athletes and those participating in sprinting and basketball appear particularly susceptible to SCD (6, 7). Data from the National Collegiate Athletic Association, for example, indicate an overall SCD incidence of 1 in 43,770 athlete-years, whereas the incidence among male basketball players increases dramatically to approximately 1 in 3,100 athlete-years (6, 7). Moreover, adolescent and young athletes between 13 and 30 years of age, especially those aged 16–21 years, are 2–2.5 times more likely to experience SCD than non-athletes (8, 9). Athletes involved in high-intensity sports are also at least 4–5 times more likely to experience SCD than those engaged in low-intensity activities (5, 10). Importantly, these fatal events occur predominantly during exercise or within the first hour after strenuous physical activity, thereby characterizing them as exercise-induced SCD (11). Consequently, identifying effective monitoring strategies and recovery interventions for young male athletes participating in high-intensity sports may play an important role in reducing the incidence of post-exercise SCD (12–15).

Rapid post-exercise recovery of heart rate (HR) and HR variability (HRV) has been strongly associated with lower risks of SCD and all-cause mortality (16, 17). Faster normalization of HR and HRV reflects more effective autonomic regulation, particularly enhanced parasympathetic or vagal reactivation after exercise (18, 19). Early recovery is characterized by restoration of vagal tone, which contributes to HR stabilization and maintenance of cardiac electrophysiological stability, both of which are critical for reducing vulnerability to malignant arrhythmias and post-exercise SCD (20). The risk of SCD is elevated during the recovery phase. Reduced HRV and delayed HR recovery (HRR), particularly within 1 min after exercise cessation, are indicators of impaired autonomic regulation and have been strongly associated with arrhythmias and SCD risk (21, 22). Specifically, HRR retains predictive power even after adjustment for conventional cardiovascular risk factors. Therefore, enhancing HRR and cardiac recovery may substantially reduce susceptibility to SCD (23). Enhancing post-exercise cardiac recovery may significantly reduce the risk of fatal cardiac events in athletes.

Music tempo, commonly quantified as beats per minute (BPM), has been shown to influence HRV, an indicator of autonomic nervous system modulation and stress-recovery status (24). The effects of BPM on HRV largely depend on its impact on sympathetic and parasympathetic activity (25, 26). High-BPM music generally stimulates sympathetic nervous system activity and may reduce HRV, particularly high-frequency (HF) components associated with parasympathetic function. In contrast, slower-tempo music tends to enhance parasympathetic activation and increase HRV (27, 28). Because rapid autonomic restoration following intense exercise is a critical physiological marker that helps prevent SCD. Carefully selected BPM music may more effectively regulate HRV, thereby enhancing post-exercise cardiac recovery (29). Previous studies have demonstrated that fast-tempo rhythmic music, typically ranging from 132 to 144 BPM, can augment cardiovascular activation and increase total HRV power during exercise, primarily through elevations in low-frequency (LF) components (27). However, such stimulation may delay parasympathetic reactivation during recovery and potentially impair timely autonomic rebalancing (30). Conversely, slower and more melodic music may be more advantageous during recovery because it enhances vagal activity, increases HF-HRV, suppresses excessive sympathetic activation, and facilitates reductions in HR (31, 32). Although low-BPM music exerts only modest effects on HRV under resting conditions, its physiological benefits appear more pronounced during recovery from high-intensity exercise (33–35). Collectively, these findings suggest that music tempo may meaningfully influence post-exercise autonomic recovery through modulation of cardiac autonomic activity (36). Therefore, the precise selection of BPM-specific music serves as a promising strategy to restore HRV and reduce SCD risk after intense physical exertion.

Recent evidence has further clarified the musical characteristics that may be most effective for enhancing repeated-sprint performance. Jebabli et al. (2023) examined preferred music during a repeated-sprint test and reported that the selected music was characterized by a fast tempo of greater than 140 beats·min^−1^ and a moderate volume of approximately 70 dB. Compared with the no-music condition, listening to preferred fast-tempo music during the repeated-sprint test improved first-set sprint performance, as reflected by a lower total sprint time (17.08 ± 0.81 vs. 17.84 ± 0.79 s; *p* = 0.006; d = 0.93), faster fastest sprint time (3.28 ± 0.17 vs. 3.39 ± 0.19 s; *p* = 0.003; d = 0.67), and lower fatigue index (1.62 ± 1.42% vs. 5.21 ± 2.62%; *p* < 0.001; d = 1.30). Moreover, listening to preferred music during the test was more effective than listening during the warm-up for improving fastest sprint time and fatigue index. Importantly, these performance benefits were not accompanied by statistically significant changes in HR responses, as no significant between-condition differences were observed for HRmean (*p* = 0.13) or HRmax (*p* = 0.18). These findings suggest that preferred fast-tempo music may enhance repeated-sprint performance without imposing an additional measurable HR burden, whereas lower-BPM music may be more relevant when the primary purpose is to facilitate post-exercise autonomic and cardiac recovery (37).

In summary, young athletes participating in high-intensity sports are at an elevated risk of SCD, particularly during the immediate post-exercise recovery period. Rapid restoration of HR and HRV is closely associated with improved autonomic stability and reduced cardiac risk. Music interventions with different BPM characteristics may modulate autonomic function and promote cardiac recovery. Therefore, it is important to distinguish between fast-tempo music used to enhance sprint performance during exercise and BPM-specific music used to optimize HR and HRV recovery after exercise. Thus, this study aims to validate and identify the optimal BPM range that enhances cardiac recovery after high-intensity exercise and to reduce the risk of SCD. The findings may offer theoretical insights and practical suggestions regarding non-pharmacological recovery strategies for athletes.

## Methods

### Participants

Ethical approval for this study was obtained from the Medical Ethics Committee of the Affiliated Hospital of Yan'an University (approval no.: IIT-G-2025072). The study was conducted in accordance with the principles of the Declaration of Helsinki. Written informed consent was obtained from all participants before participation. In addition, a pilot intervention phase was conducted before the formal experiment to verify the feasibility and procedural stability of the study protocol.

The inclusion criteria were male collegiate athletes officially registered at their respective universities as of September 1, 2024, who voluntarily agreed to participate and provided written informed consent. The exclusion criteria included a history of congenital heart disease, liver disease, cardiovascular disease, or any other medical condition that could influence performance during the high-intensity anaerobic cycling test. Participants who had consumed alcohol or stimulant-containing beverages within 24 h before testing were also excluded. Before inclusion, all participants underwent a physical examination at the Affiliated Hospital of Yan'an University in Yan'an City, Shaanxi Province, China, to confirm eligibility.

Initially, 300 male collegiate sprinters were randomly selected from a sampling pool of 1,000 male student athletes registered at Chinese universities as of September 1, 2024. Recruitment advertisements were disseminated through social media platforms, including WeChat, QQ, and Weibo. All potential participants received detailed study information by email at least 7 days before enrollment. Upon arrival at the laboratory, participants received an additional verbal explanation of the study procedures and subsequently provided written informed consent before participation.

Among the 300 initially selected athletes, 265 participants met all eligibility criteria and completed the study protocol, and their data were included in the final statistical analysis. The remaining 35 participants were excluded because of failure to meet the eligibility criteria, withdrawal before study completion, violation of pre-test requirements, or incomplete or unusable physiological data. Accordingly, all demographic, anthropometric, HR, and HRV analyses were conducted using the final valid sample of 265 participants.

Sample size estimation was incorporated into the “Participants” section to present participant recruitment, eligibility, final sample size, and statistical power in a unified manner. An *a priori* sample size calculation was performed using G*Power software. Because the study compared multiple experimental conditions, the required sample size was estimated for a one-way analysis of variance involving 10 groups. Assuming a medium effect size (f = 0.25), an alpha level of 0.05, and a desired statistical power of 0.80, the minimum required total sample size was calculated to be 260 participants. The final valid sample of 265 participants exceeded this threshold and therefore provided adequate statistical power for the primary between-group analyses.

### Procedure

#### Collection of baseline data

After eligibility screening and written informed consent, baseline demographic and anthropometric data were collected for all eligible participants. Resting HRV was also measured before the intervention to establish baseline physiological status and evaluate comparability among groups.

#### Grouping

After eligibility screening, informed consent, baseline demographic and anthropometric assessment, and resting HRV measurement, the 265 eligible participants were randomly allocated to one of 10 experimental conditions, consisting of one no-music control group and nine music-tempo intervention groups. Randomization was performed using a computer-generated random-number sequence. The allocation sequence was generated before the intervention by an investigator who was not involved in HRV assessment or outcome analysis. No stratified randomization procedure was applied. Participants were assigned sequentially according to enrollment order and the corresponding random allocation code. The control group received no music during the recovery period, whereas the intervention groups listened to music at different BPM levels during post-exercise recovery. Group allocation was as follows: control group, *n* = 27; 50 BPM group, *n* = 30; 60 BPM group, *n* = 24; 70 BPM group, *n* = 25; 80 BPM group, *n* = 26; 90 BPM group, *n* = 25; 100 BPM group, *n* = 28; 110 BPM group, *n* = 28; 120 BPM group, *n* = 26; and 130 BPM group, *n* = 26.

Subsequently, one-way analysis of variance (ANOVA) was performed solely to evaluate baseline comparability among groups, including anaerobic cycling performance and baseline HRV variables. Importantly, the randomization results were not modified on the basis of statistical significance testing. The baseline analysis was conducted exclusively to confirm descriptive comparability across groups rather than to influence participant allocation.

#### Experimental controls

To minimize potential confounding factors, all participants were required to avoid alcohol and stimulant-containing beverages within 24 h before testing. Eligibility was confirmed through a pre-test physical examination, and participants with medical conditions that could influence high-intensity anaerobic cycling performance were excluded. The same experimental protocol was applied across groups, with the only difference being whether participants received no music or music at different BPM levels during the post-exercise recovery period.

#### Experimental procedure and participant flow

**Figure 1:**
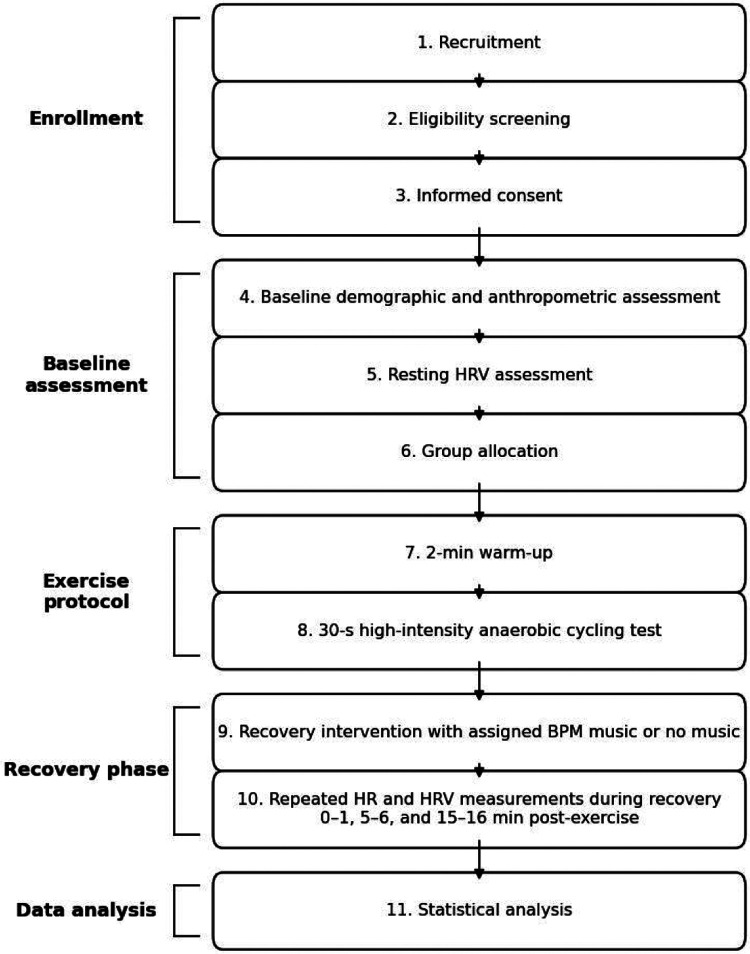
Flowchart of the study protocol.

To improve methodological clarity and reproducibility, a CONSORT-style participant flowchart was included as Figure 1. The flowchart illustrates the sequential process of participant recruitment, screening, allocation, intervention, and final analysis. Briefly, 1,000 registered male student athletes were screened as the initial sampling pool, from which 300 male collegiate sprinters were randomly selected and assessed for eligibility. Before final analysis, 35 participants were excluded because of ineligibility, withdrawal, violation of pre-test requirements, or incomplete or unusable physiological data. Consequently, 265 participants were included in the final statistical analysis. The subsequent experimental protocol included informed consent acquisition, baseline demographic and anthropometric assessment, resting HRV measurement, random group allocation, warm-up, completion of the high-intensity anaerobic cycling test, post-exercise music intervention or a no-music recovery condition, repeated HR and HRV measurements during recovery, and final statistical analysis.

### Baseline data collection

Demographic and anthropometric data were collected by two trained evaluators. Variables included age, sex, height, body weight, and body mass index, which were measured using the InBody 770 body composition analyzer.

### Anaerobic cycling test

The anaerobic cycling test was conducted by two trained assessors. Before testing, all participants received standardized instructions regarding test procedures and safety precautions. Each participant first completed a 2-min warm-up cycling session, followed by a 30-s bout of high-intensity anaerobic cycling. The resistance load was standardized at 0.083 kp/kg body weight. A Polar chest-strap HR monitor (Polar, Finland) was used throughout testing to ensure that HR exceeded 95% of the estimated maximal HR during exercise. The resulting exercise performance data were subsequently used for analysis.

The cycling protocol used in the present study was based on the core principles of the traditional Wingate anaerobic test because it incorporated a 30-s maximal cycling effort and a body-mass-adjusted resistance load. However, the protocol did not constitute a fully standardized Wingate anaerobic test performed with a dedicated Wingate testing system capable of generating conventional Wingate-derived parameters, such as peak power, mean power, and fatigue index. Instead, the protocol was designed primarily to provide a standardized high-intensity anaerobic exercise stimulus before the recovery intervention. To avoid methodological ambiguity, the procedure is therefore described throughout the manuscript as a “high-intensity anaerobic cycling test” or “Wingate-style anaerobic cycling test.”

### HR and HRV Test

HRV assessments were conducted by two trained professional assessors who explained all testing procedures to the participants before data collection. To ensure methodological consistency, repeatability, and clinical reliability, HRV measurements strictly adhered to the standards established by the European Society of Cardiology and the North American Society of Pacing and Electrophysiology. Prior to analysis, ectopic beats and artifact signals were identified and excluded to improve data quality. Participants remained seated quietly for 5 min before baseline assessment to acclimatize to the testing environment. During this baseline period, breathing was paced at 12–20 breaths/min to reduce respiratory interference with HRV measurements. This breathing control procedure was applied only during the resting baseline assessment. During the post-exercise recovery phase, participants were instructed to remain seated quietly and breathe naturally; however, respiratory rate and tidal volume were not continuously monitored or statistically controlled during recovery. If the resting HR exceeded 85 beats/min, the assessment was repeated to minimize the influence of incomplete physiological stabilization. HRV was measured using the Biospace Ubpulse T1 system (Biospace, South Korea). Baseline HRV data were collected during the 1-min period immediately preceding the warm-up session. During the recovery phase (0–16 min), participants wore Sony WH-1000XM3 headphones (Sony, Japan) and listened to their assigned music intervention at tempos ranging from 50 to 130 BPM. HRV measurements were obtained during three predefined recovery windows: 0–1 min, 5–6 min, and 15–16 min after exercise. The previous description referring to “four time points” was corrected because only three post-exercise recovery windows were included in the present analysis. Detailed HRV parameters are presented in Table 1.

**Table 1:** Heart rate variability index parameters.

Heart rate variability indicator	Definition	Parameter meaning
HR (beats/min)	Average heart rate	Reflects the mean RR interval
SDNN (ms)	Standard deviation of all normal RR intervals	General indicator of autonomic nervous system activity
LF (ms^2^)	Low-frequency power	Traditionally associated with mixed sympathetic and parasympathetic modulation
HF (ms^2^)	High-frequency power	Primarily reflects parasympathetic nervous system activity
LFnorm (n.u.)	LF/(LF + HF) × 100	Reflects the relative contribution of LF activity
HFnorm (n.u.)	HF/(LF + HF) × 100	Reflects the relative contribution of parasympathetic activity
LF/HF	Low-frequency power/high-frequency power	Traditionally interpreted as sympathovagal balance
HRV index	HRV index	Reflects cycle-to-cycle HRV variation

Because autonomic recovery changes rapidly immediately after high-intensity exercise, 1-min HRV windows were selected to capture short-term dynamic fluctuations during the early recovery phase while minimizing participant burden. This design enabled assessment of rapid post-exercise autonomic responses; however, the absence of an intermediate recovery measurement, such as at 10 min, limited characterization of the complete temporal recovery trajectory between the early and late recovery stages. Accordingly, the present findings should be interpreted as observations from discrete recovery windows rather than as a continuous autonomic recovery profile. In addition, frequency-domain HRV indices derived from ultra-short-term recordings, particularly LF, HF, LFnorm, HFnorm, and LF/HF, may demonstrate lower reliability than values derived from conventional 5-min recordings. This limitation is important because the 1996 Task Force recommendations indicate that short-term frequency-domain HRV analysis is conventionally based on recordings of approximately 5 min in duration. Consequently, 1-min recordings represent the lower acceptable boundary for spectral estimation, especially for LF-related parameters, and may not provide fully stable frequency-domain measurements. Another important consideration is that respiration was not continuously monitored during the recovery phase. Variations in respiratory rate or breathing depth may therefore have influenced HF- and HFnorm-related outcomes. This issue is particularly relevant because music tempo may entrain or alter breathing patterns, and tempo-related respiratory changes may partly explain the observed differences in respiration-sensitive HRV indices among BPM conditions. Therefore, frequency-domain HRV parameters obtained from the 1-min recovery windows were interpreted cautiously and were considered supportive rather than definitive indicators of autonomic modulation. Interpretation of post-exercise autonomic recovery was therefore based primarily on HR and time-domain HRV measures, particularly SDNN, together with the overall recovery pattern across time points. HF, HFnorm, and LF/HF were interpreted as secondary and exploratory indices because of their sensitivity to respiratory variation and the absence of respiratory monitoring during recovery. Future studies should consider using conventional 5-min HRV recordings when feasible and incorporating intermediate recovery measurements, such as at 10 min, to better characterize the temporal progression of autonomic recovery. Alternatively, validated time-domain or non-linear HRV indicators may be more appropriate for real-time or ultra-short-term post-exercise monitoring. Future investigations should also include continuous respiratory monitoring using respiratory belts or flow sensors and should statistically adjust for respiratory rate and tidal volume when analyzing frequency-domain HRV parameters during recovery.

### Outcome measures

To reduce ambiguity in data interpretation and minimize the risk of selective outcome reporting, outcome measures were clearly predefined in the revised manuscript. The primary outcomes were post-exercise HR recovery and SDNN during the recovery phase. HR recovery was selected because it directly reflects the rate of HR decline following high-intensity exercise and is closely associated with short-term autonomic recovery. SDNN was selected as the primary HRV outcome because it is a time-domain HRV parameter that can be interpreted more appropriately within short-term recovery windows than frequency-domain indices derived from ultra-short-term recordings.

Secondary outcomes included LF, HF, LFnorm, HFnorm, LF/HF, and HRV index during recovery. These frequency-domain and composite HRV parameters were used to support interpretation of autonomic recovery patterns. However, because these indices were derived from 1-min ultra-short-term HRV recordings and may be influenced by respiratory variation, they were interpreted as secondary and exploratory outcomes rather than as stand-alone evidence of autonomic modulation.

The primary statistical analysis focused on the time × group interaction effects for HR and SDNN to determine whether the recovery trajectories differed among BPM conditions. Secondary analyses examined time × group interaction effects and Bonferroni-adjusted *post hoc* comparisons for LF, HF, LFnorm, HFnorm, LF/HF, and HRV index.

### Music BPM settings

Two professional music producers used FL Studio to modify Blue and White Porcelain by Jay Chou into instrumental tracks with tempos of 50, 60, 70, 80, 90, 100, 110, 120, and 130 BPM. These edited tracks were used during the post-exercise recovery phase.

The same instrumental composition was used across all BPM conditions to isolate the influence of tempo while maintaining relative consistency in melody, harmony, timbre, and overall musical structure. Pitch-preserving tempo modification techniques were applied during audio editing to minimize unintended alterations in tonal characteristics resulting from tempo adjustment. All tracks were played at a standardized sound intensity of 60 dB during recovery.

Nevertheless, the use of a single musical composition could not completely eliminate the emotional influence of music. In addition to BPM, factors such as musical style, melody, harmony, familiarity, and individual preference may affect emotional responses and autonomic regulation. Participants’ familiarity with or preference for “Blue and White Porcelain” was not assessed in the present study. Therefore, the findings should be interpreted specifically as the effect of tempo manipulation within a single instrumental music context rather than as evidence of a universal BPM effect across different musical genres or styles. Future studies should incorporate multiple songs representing different musical styles at equivalent BPM levels and should quantitatively assess music preference, familiarity, emotional valence, arousal, and psychological responses before and after intervention.

### Experimental controls

Before testing, all participants received standardized instructions regarding experimental procedures, precautions, and testing arrangements. The testing environment was maintained at a temperature of 23 °C and relative humidity of 65%, with minimal ambient noise. All assessments were conducted between 3:00 PM and 5:00 PM to minimize circadian variation, and no more than six participants were tested per day. Participants were instructed to avoid listening to music, engaging in vigorous physical activity, and consuming food or caffeinated beverages within 3 h before testing. Alcohol consumption was prohibited for at least 48 h before assessment.

During the recovery period, music was played continuously at 60 dB. This sound intensity was selected to provide a clearly audible yet non-startling listening environment during recovery. A sound level of approximately 60 dB is comparable to normal conversational intensity and remains below commonly cited safe listening thresholds (38). Previous music-intervention studies have similarly used moderate listening levels, typically between 50 and 60 dB, to minimize participant discomfort and excessive auditory stimulation while ensuring that the music remained clearly perceptible (39, 40). Furthermore, previous studies examining auditory influences on HRV have used sound intensities near 60 dB, supporting the appropriateness of this listening level for investigations involving autonomic responses (41). Therefore, a standardized sound intensity of 60 dB was used across all BPM conditions to minimize the possibility that loudness itself could confound HR or HRV responses. All experimental data were securely recorded and stored.

Although environmental temperature, humidity, testing time, sound intensity, pre-test diet, caffeine intake, alcohol intake, and physical activity were controlled, participants’ psychological responses to the assigned music were not directly evaluated. This issue has been added as a methodological limitation and should be addressed in future research using validated instruments assessing music preference, familiarity, emotional valence, and arousal.

### Data entry and statistical analysis

Data entry was independently performed by two researchers. Missing data representing less than one-third of the dataset were replaced using mean imputation, whereas datasets with missing values exceeding this threshold were excluded from analysis. Data were organized using Microsoft Excel and statistically analyzed using SPSS version 26.0. Descriptive statistics for demographic characteristics are presented as frequencies and percentages, whereas continuous variables with normal distributions are expressed as mean ± standard deviation.

To reduce ambiguity in interpretation and minimize the possibility of selective outcome reporting, outcome measures were specified before the revised statistical analysis. The primary outcomes were post-exercise HR recovery and SDNN during recovery. HR recovery was selected because it directly reflects the rate of post-exercise HR decline following high-intensity exercise. SDNN was selected as the primary HRV outcome because it is a time-domain HRV measure that is more appropriate for short-duration recovery windows than frequency-domain indices derived from ultra-short-term recordings. Secondary outcomes included LF, HF, LFnorm, HFnorm, LF/HF, and HRV index. These parameters were used to support interpretation of autonomic recovery patterns; however, they were interpreted cautiously because they were derived from 1-min ultra-short-term HRV recordings and may have been influenced by respiratory variation.

Baseline comparability among BPM groups for demographic characteristics, anaerobic cycling performance, and resting HRV indices was evaluated using one-way ANOVA. To compare HR and HRV responses during recovery across groups and time points, two-way repeated-measures ANOVA was performed, with group as the between-subject factor and time as the within-subject factor. The primary analysis focused on the time × group interaction effects for HR and SDNN. Secondary analyses examined time × group interaction effects and Bonferroni-adjusted *post hoc* comparisons for LF, HF, LFnorm, HFnorm, LF/HF, and HRV index. When significant main effects or interaction effects were identified, Bonferroni-adjusted *post hoc* pairwise comparisons were conducted to control for multiple testing across the 10 BPM groups. Only findings that remained significant after Bonferroni correction were considered statistically significant. A *p*-value <0.05 was considered statistically significant.

## Results

### Baseline characteristics

A total of 265 participants were included in the final dataset. Their demographic and physical characteristics are presented in Table 2.

**Table 2:** Physical characteristics of the 256 participants.

Participants (*N* = 265)	Age (years)	Height (cm)	Weight (kg)	BMI (kg/m^2^)
A (27)	20.15 ± 0.72	171.11 ± 7.71	69.30 ± 14.44	23.39 ± 3.93
B (30)	20.38 ± 2.06	174.17 ± 4.60	71.13 ± 8.32	23.42 ± 2.66
C (24)	20.21 ± 0.78	170.21 ± 7.27	67.08 ± 11.53	22.93 ± 3.43
D (25)	20.16 ± 0.75	170.28 ± 7.33	67.80 ± 11.58	23.15 ± 3.41
E (26)	20.15 ± 0.73	170.77 ± 7.65	68.92 ± 14.60	23.35 ± 4.01
F (25)	20.68 ± 1.93	172.42 ± 6.58	68.42 ± 11.10	23.01 ± 2.84
G (28)	20.54 ± 1.60	172.38 ± 6.44	69.90 ± 12.34	23.35 ± 3.05
H (28)	21.00 ± 1.85	172.85 ± 4.69	68.60 ± 9.28	22.85 ± 2.77
I (26)	20.19 ± 0.75	170.77 ± 7.65	69.26 ± 14.48	23.48 ± 3.98
J (26)	20.15 ± 0.73	170.77 ± 7.65	68.92 ± 14.60	23.35 ± 4.01

A, control group; B, 50 bpm group; C, 60 bpm group; D, 70 bpm group; E, 80 bpm group; F, 90 bpm group; G, 100 bpm group; H, 110 bpm group; I, 120 bpm group; J, 130 bpm group.

### Analysis of pre-exercise HR and HRV indicators

As shown in Table 3, there were no statistically significant differences in HR and HRV indicators among the groups before high-intensity exercise (*p* > 0.05).

**Table 3:** Analysis of pre-exercise HRV indicators.

Index	Groups	(mean ± SD)	Groups	(mean ± SD)	F	P
HR (bpm)	A (27)	78.30 ± 10.54	F (25)	75.88 ± 13.49	1.243	0.269
	B (30)	77.19 ± 12.92	G (28)	74.75 ± 12.90		
	C (24)	80.50 ± 12.06	H (28)	73.00 ± 10.69		
	D (25)	77.68 ± 13.87	I (26)	79.85 ± 12.20		
	E (26)	79.35 ± 11.62	J (26)	81.31 ± 11.87		
SDNN (ms)	A (27)	62.67 ± 8.63	F (25)	59.71 ± 10.81	0.926	0.503
	B (30)	57.84 ± 20.50	G (28)	60.79 ± 14.98		
	C (24)	64.67 ± 15.52	H (28)	55.71 ± 15.77		
	D (25)	62.20 ± 18.06	I (26)	60.92 ± 12.82		
	E (26)	60.81 ± 19.06	J (26)	65.81 ± 20.15		
LF (ms^2^)	A (27)	7.17 ± 0.32	F (25)	6.89 ± 0.47	1.57	0.124
	B (30)	6.76 ± 1.05	G (28)	7.00 ± 0.83		
	C (24)	7.02 ± 0.44	H (28)	6.69 ± 0.78		
	D (25)	6.92 ± 0.63	I (26)	7.09 ± 0.37		
	E (26)	7.07 ± 0.46	J (26)	7.07 ± 0.41		
HF (ms^2^)	A (27)	6.59 ± 0.99	F (25)	6.13 ± 0.47	0.766	0.648
	B (30)	6.24 ± 0.41	G (28)	6.27 ± 1.08		
	C (24)	6.41 ± 0.89	H (28)	6.18 ± 0.77		
	D (25)	6.38 ± 1.13	I (26)	6.48 ± 0.93		
	E (26)	6.36 ± 0.75	J (26)	6.52 ± 1.14		
LFnorm (n.u.)	A (27)	53.47 ± 1.95	F (25)	52.69 ± 3.89	1.083	0.376
	B (30)	53.44 ± 3.70	G (28)	52.89 ± 3.84		
	C (24)	52.99 ± 2.86	H (28)	52.05 ± 3.33		
	D (25)	53.66 ± 3.09	I (26)	54.04 ± 2.36		
	E (26)	53.02 ± 2.57	J (26)	54.11 ± 3.09		
HFnorm (n.u.)	A (27)	46.53 ± 1.95	F (25)	47.31 ± 3.89	1.069	0.386
	B (30)	46.56 ± 3.70	G (28)	47.11 ± 3.84		
	C (24)	46.59 ± 3.76	H (28)	48.02 ± 3.32		
	D (25)	46.34 ± 3.09	I (26)	45.97 ± 2.36		
	E (26)	46.98 ± 2.57	J (26)	45.89 ± 3.09		
HRVindex	A (27)	17.97 ± 4.36	F (25)	15.85 ± 2.97	1.322	0.226
	B (30)	15.83 ± 5.31	G (28)	15.14 ± 5.26		
	C (24)	17.50 ± 6.30	H (28)	15.36 ± 3.38		
	D (25)	16.86 ± 4.98	I (26)	17.99 ± 5.76		
	E (26)	16.77 ± 4.73	J (26)	17.86 ± 6.01		

A, control group; B, 50 bpm group; C, 60 bpm group; D, 70 bpm group; E, 80 bpm group; F, 90 bpm group; G, 100 bpm group; H, 110 bpm group; I, 120 bpm group; J, 130 bpm group.

### Analysis of anaerobic performance during high-intensity exercise

Table 4 confirms that there were no statistically significant differences in anaerobic performance indicators during the exercise across the groups (*p* > 0.05).

**Table 4:** Anaerobic performance indicators during high-intensity exercise.

Index	Groups	(mean ± SD)	Groups	(mean ± SD)	F	P
MP(W)	A (27)	627.57 ± 58.25	F (25)	587.7 ± 165.5	1.373	0.201
	B (30)	653.29 ± 90.11	G (28)	597.8 ± 163.4		
	C (24)	612.71 ± 70.22	H (28)	585.6 ± 164.7		
	D (25)	589.02 ± 52.22	I (26)	571.0 ± 90.8		
	E (26)	586.80 ± 79.73	J (26)	612.1 ± 96.4		
MW(W/kg)	A (27)	9.38 ± 1.93	F (25)	8.55 ± 1.99	0.926	0.503
	B (30)	9.26 ± 1.37	G (28)	8.51 ± 1.85		
	C (24)	9.48 ± 2.35	H (28)	8.44 ± 1.98		
	D (25)	9.00 ± 2.05	I (26)	8.57 ± 2.23		
	E (26)	8.89 ± 2.30	J (26)	9.24 ± 2.47		
PP(W)	A (27)	936.37 ± 68.74	F (25)	892.44 ± 263.16	0.718	0.692
	B (30)	987.53 ± 188.43	G (28)	933.25 ± 272.66		
	C (24)	950.65 ± 63.14	H (28)	917.14 ± 308.42		
	D (25)	925.08 ± 53.77	I (26)	890.38 ± 89.76		
	E (26)	911.07 ± 107.57	J (26)	935.61 ± 87.44		
PW(W/kg)	A (27)	13.97 ± 2.59	F (25)	12.94 ± 2.79	0.882	0.542
	B (30)	13.98 ± 2.61	G (28)	13.24 ± 2.79		
	C (24)	14.66 ± 3.08	H (28)	13.12 ± 3.49		
	D (25)	14.08 ± 2.86	I (26)	13.32 ± 2.75		
	E (26)	13.75 ± 3.10	J (26)	14.16 ± 3.43		
FI(%)	A (27)	14.44 ± 1.70	F (25)	14.44 ± 4.98	1.083	0.376
	B (30)	16.30 ± 3.31	G (28)	15.32 ± 5.07		
	C (24)	15.05 ± 2.28	H (28)	15.29 ± 5.96		
	D (25)	14.47 ± 1.37	I (26)	13.94 ± 2.16		
	E (26)	14.64 ± 2.48	J (26)	14.59 ± 2.52		
TW(J)	A (27)	18827.05 ± 1747.46	F (25)	17629.92 ± 4965.13	1.373	0.201
	B (30)	19598.63 ± 2703.23	G (28)	17932.93 ± 4901.80		
	C (24)	18381.20 ± 2106.74	H (28)	17566.71 ± 4941.61		
	D (25)	17670.50 ± 1566.59	I (26)	17128.54 ± 2723.73		
	E (26)	17604.06 ± 2391.79	J (26)	18363.64 ± 2893.35		

MP, mean power; W; MW, mean power relative to body mass, W/kg; PP, peak power; W; PW, peak power relative to body mass, W/kg; FI, fatigue index, %; TW, total work, J. MP was calculated as the average power output during the 30-s high-intensity anaerobic cycling test. PP was defined as the highest power output recorded during the test. MW and PW were calculated by dividing MP and PP by body mass, respectively. FI was calculated as [(PP−minimum power)/PP] × 100. TW was calculated as the total mechanical work completed during the 30-s test, that is, mean power × exercise duration. *p < 0.05.A: control group; B: 50 bpm group; C: 60 bpm group; D: 70 bpm group; E: 80 bpm group; F: 90 bpm group; G: 100 bpm group; H: 110 bpm group; I: 120 bpm group; J: 130 bpm group.

### Effects of BPM Music on HR and HRV during the recovery phase

**Figure 2:**
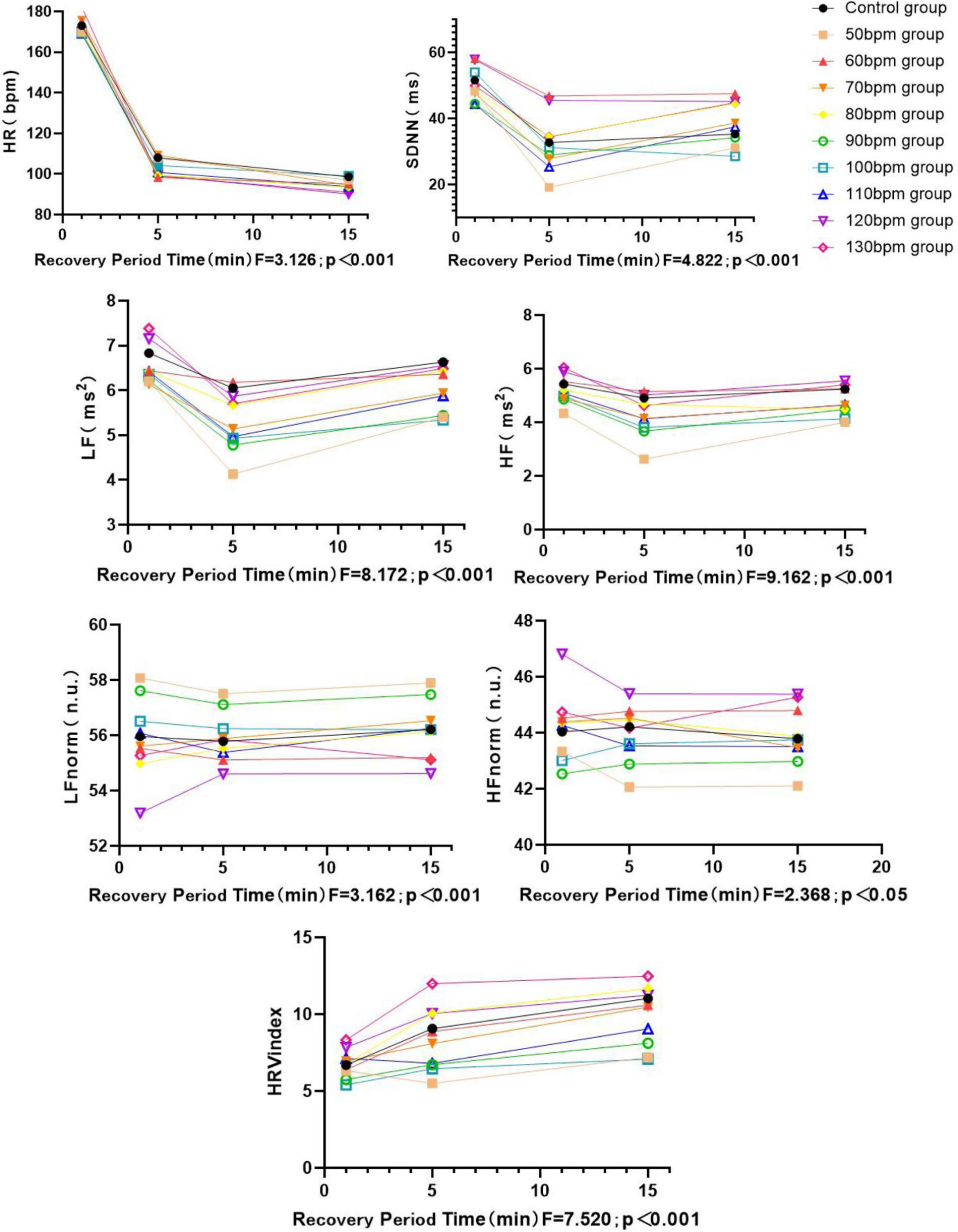
Differences in HR and HRV during Recovery across Different BPM.

Table 5 and Figure 2 presents HR and HRV indices during the post-exercise recovery period across different BPM conditions. Overall, HR decreased substantially from 0 to 1 min to 15–16 min after exercise, whereas HRV indices demonstrated variable recovery patterns over time.

**Table 5:** Differences in HR and HRV during Recovery across Different BPM Levels.

Groups	RP	HR (bpm)	SDNN (ms)	LF (ms2)	HF (ms2)	LFnorm (n.u.)	HFnorm (n.u.)	HRVindex
A (27)	1	173.04 ± 11.68	51.56 ± 27.34	6.83 ± 1.62	5.43 ± 1.82	55.96 ± 3.29	44.04 ± 3.29	6.69 ± 5.04
	5	108.00 ± 4.39	32.74 ± 11.57	6.05 ± 1.40	4.91 ± 1.49	55.78 ± 3.99	44.22 ± 3.99	9.07 ± 2.89
	15	98.63 ± 6.14	35.26 ± 8.45	6.63 ± 1.27	5.24 ± 1.39	56.21 ± 3.17	43.79 ± 3.17	11.03 ± 2.95
B (30)	1	169.68 ± 1.75	48.30 ± 25.38	6.20 ± 1.51	4.34 ± 1.61	58.08 ± 3.67	43.33 ± 3.79	6.33 ± 1.94
	5	107.69 ± 8.00	19.16 ± 11.93	4.13 ± 1.38	2.63 ± 1.59	57.50 ± 4.97	42.06 ± 6.29	5.51 ± 2.03
	15	97.03 ± 12.85	31.12 ± 26.03	5.40 ± 1.23	4.00 ± 1.12	57.90 ± 4.45	42.10 ± 4.45	7.19 ± 3.37
C (24)	1	175.00 ± 11.78	58.13 ± 18.15	6.44 ± 1.93	5.52 ± 2.35	55.53 ± 6.34	44.52 ± 6.38	6.39 ± 4.07
	5	98.21 ± 10.95	46.83 ± 23.79	6.18 ± 1.61	5.15 ± 1.59	55.11 ± 5.37	44.77 ± 5.43	8.88 ± 4.16
	15	95.13 ± 12.24	47.54 ± 20.71	6.36 ± 1.34	5.25 ± 1.47	55.20 ± 3.85	44.80 ± 3.85	10.60 ± 3.32
D (25)	1	175.56 ± 12.27	47.64 ± 12.73	6.13 ± 1.60	4.89 ± 1.31	55.61 ± 4.73	44.39 ± 4.73	6.94 ± 3.17
	5	109.28 ± 8.14	27.84 ± 13.05	5.14 ± 1.32	4.15 ± 1.40	55.88 ± 4.74	44.52 ± 4.96	8.09 ± 3.20
	15	94.48 ± 11.95	38.60 ± 18.14	5.94 ± 1.19	4.64 ± 1.32	56.53 ± 4.10	43.47 ± 4.10	10.47 ± 3.33
E (26)	1	173.31 ± 9.06	48.12 ± 16.56	6.43 ± 2.00	5.20 ± 2.11	54.98 ± 6.39	44.34 ± 5.72	6.77 ± 3.71
	5	99.38 ± 14.04	34.58 ± 18.36	5.67 ± 1.06	4.67 ± 1.44	55.53 ± 5.20	44.47 ± 5.20	10.07 ± 4.87
	15	93.50 ± 12.94	44.46 ± 19.63	6.43 ± 1.17	4.48 ± 1.52	56.13 ± 5.52	43.87 ± 5.52	11.68 ± 4.75
F (25)	1	169.42 ± 1.64	44.46 ± 25.48	6.22 ± 1.55	4.86 ± 1.60	57.62 ± 3.81	42.54 ± 4.18	5.76 ± 1.42
	5	99.32 ± 12.83	28.96 ± 14.80	4.78 ± 1.51	3.67 ± 1.48	57.12 ± 7.29	42.88 ± 7.29	6.72 ± 2.42
	15	93.64 ± 12.18	34.16 ± 11.66	5.44 ± 1.32	4.48 ± 1.52	57.48 ± 5.91	42.98 ± 5.67	8.12 ± 3.30
G (28)	1	169.54 ± 1.36	53.99 ± 26.05	6.36 ± 1.56	4.98 ± 1.27	56.51 ± 5.00	43.01 ± 4.62	5.41 ± 1.43
	5	104.21 ± 10.81	31.20 ± 16.08	4.93 ± 1.69	3.81 ± 1.72	56.24 ± 6.31	43.61 ± 6.18	6.45 ± 2.18
	15	99.04 ± 9.90	28.55 ± 16.98	5.34 ± 0.91	4.14 ± 1.12	56.20 ± 6.23	43.75 ± 6.22	7.08 ± 2.00
H (28)	1	169.15 ± 1.57	44.40 ± 20.07	6.42 ± 1.24	5.08 ± 1.24	56.08 ± 4.28	44.27 ± 4.34	7.13 ± 4.65
	5	100.79 ± 12.03	25.36 ± 10.71	4.97 ± 1.41	4.14 ± 1.50	55.38 ± 6.61	43.54 ± 7.65	6.81 ± 2.52
	15	93.75 ± 13.58	37.46 ± 15.02	5.88 ± 1.19	4.65 ± 1.32	56.25 ± 4.54	43.50 ± 4.51	9.06 ± 3.93
I (26)	1	170.12 ± 11.59	57.85 ± 24.06	7.15 ± 1.47	5.89 ± 2.16	53.19 ± 7.86	46.81 ± 7.86	7.84 ± 6.29
	5	99.27 ± 12.63	45.46 ± 16.84	5.87 ± 1.51	5.02 ± 1.68	54.60 ± 4.48	45.40 ± 4.48	10.04 ± 5.38
	15	90.15 ± 9.08	45.12 ± 19.41	6.55 ± 1.05	5.55 ± 1.50	54.62 ± 3.76	45.38 ± 3.76	11.24 ± 4.53
J (26)	1	182.38 ± 18.94	49.92 ± 15.94	7.38 ± 1.51	6.05 ± 1.54	55.27 ± 2.29	44.74 ± 2.29	8.33 ± 3.53
	5	98.96 ± 10.27	34.42 ± 16.94	5.70 ± 1.31	4.62 ± 1.50	55.84 ± 5.42	44.17 ± 5.42	11.99 ± 8.90
	15	91.00 ± 11.10	44.77 ± 19.14	6.49 ± 1.13	5.40 ± 1.28	55.12 ± 3.43	45.27 ± 3.48	12.48 ± 4.29
F value between groups		3.126	4.822	8.172	9.162	3.162	2.368	7.520
Time*Group		F (20.302, 548.149) = 2.245, *p* = 0.001, *η*p^2^ = 0.077	F (24.050, 686.753) = 1.336, *p* = 0.131, *η*p^2^ = 0.045	F (22.617, 645.838) = 1.630，*p* = 0.033，*η*p^2^ = 0.054	F (23.220, 663.065) = 1.644, *p* = 0.029, *η*p^2^ = 0.054	F (22.366, 638.667) = 0.853, *p* = 0.660, *η*p^2^ = 0.029	F (23.597, 673.819) = 0.913, *p* = 0.583, *η*p^2^ = 0.031	F (22.977, 656.127) = 1.317, *p* = 0.147, *η*p^2^ = 0.044
Bonferroni P		.029	<0.001	<.001	<.001	.023	.043	<.001
Post hoc		A > C, E, F, H, I; I < A, B, D, G, J	A < C, I; I > A, B, D, E, F, G, H, J	A > B, D, F, H, G; G < A, C, E, H, J	A > B, D, F, G, H; I > B, D, F, G, H	A > I; I < A, B, D, F, G, H	A < I; I > A, B, F, G, H	A < I, J; J, I > A, B, C, D, E, F, G, H

RP, recovery period. Time (min). Data are presented as mean ± SD. A, control group; B, 50 BPM group; C, 60 BPM group; D, 70 BPM group; E, 80 BPM group; F, 90 BPM group; G, 100 BPM group; H, 110 BPM group; I, 120 BPM group; J, 130 BPM group. Group differences were examined using repeated-measures ANOVA. Bonferroni correction was applied for *post-hoc* pairwise comparisons among groups. Only Bonferroni-adjusted comparisons with adjusted *p* < 0.05 were considered statistically significant. Effect sizes were reported as partial eta squared ηp^2^ for ANOVA effects and Cohen's d for pairwise comparisons.

The two-way repeated-measures ANOVA revealed a significant time × group interaction for HR, F(20.302, 548.149) = 2.245, *p* = 0.001, *η*p^2^ = 0.077, indicating that HR recovery trajectories differed significantly among the BPM groups. Significant time × group interactions were also observed for LF, F(22.617, 645.838) = 1.630, *p* = 0.033, *η*p^2^ = 0.054 and HF, F(23.220, 663.065) = 1.644, *p* = 0.029, *η*p^2^ = 0.054, suggesting that temporal changes in selected frequency-domain HRV parameters varied according to BPM condition.

In contrast, no significant time × group interactions were observed for SDNN, F(24.050, 686.753) = 1.336, *p* = 0.131, *η*p^2^ = 0.045; LFnorm, F(22.366, 638.667) = 0.853, *p* = 0.660, *η*p^2^ = 0.029; HFnorm, F(23.597, 673.819) = 0.913, *p* = 0.583, *η*p^2^ = 0.031; or HRV index, F(22.977, 656.127) = 1.317, *p* = 0.147, *η*p^2^ = 0.044. These findings indicate that the recovery trajectories of these parameters did not differ significantly among BPM groups.

Bonferroni-corrected *post hoc* analyses identified significant between-group differences in several HR and HRV parameters. Overall, the 120 BPM group demonstrated a relatively favorable recovery profile, particularly with respect to HR recovery and selected frequency-domain HRV indices. However, this pattern should not be interpreted as evidence of consistent superiority of 120 BPM across all autonomic markers. Notably, the 120 BPM condition was not superior to the 130 BPM condition across all reported outcomes. At 15 min of recovery, the HRV index in the 130 BPM group was 12.48 ± 4.29, which was comparable to, and numerically higher than, the value observed in the 120 BPM group (11.24 ± 4.53). Furthermore, the absence of a significant time × group interaction for HRV index indicates that HRV index recovery trajectories did not differ significantly among BPM conditions.

Taken together, within the BPM range and experimental conditions examined in the present study, 120 BPM music appeared to be associated with a generally more favorable post-exercise recovery tendency, primarily reflected by improved HR recovery and selected frequency-domain HRV responses. Nevertheless, because significant time × group interactions were observed only for HR, LF, and HF, but not for SDNN, LFnorm, HFnorm, or HRV index, the findings should be interpreted as evidence of a partially tempo-dependent recovery response rather than definitive evidence that 120 BPM is universally optimal or consistently superior. The comparable HRV index values observed in the 130 BPM group further suggest that 130 BPM may confer similar benefits for certain HRV-related recovery outcomes.

## Discussion

SCD after high-intensity physical exertion remains a major concern in sports medicine and athlete safety, particularly among young individuals participating in strenuous activity (42). The immediate post-exercise period represents a transient phase of heightened cardiovascular vulnerability, during which susceptibility to SCD markedly increases. Increasing attention has therefore been directed toward the relationship between exercise-induced sudden death, defined as sudden death occurring during or shortly after physical activity, and HRV in both sports medicine and cardiovascular health domains (43). HRV reflects the autonomic regulation of cardiac function and has been widely used in predicting cardiac health and physiological stress responses (44–47). Understanding HRV dynamics holds promise in predicting and preventing exercise-related sudden death.

Music-based interventions have emerged as a practical non-pharmacological approach for modulating psychological and physiological responses during recovery (33, 48). Among the various musical characteristics, tempo, commonly quantified in BPM, has received substantial attention in music therapy and psychophysiological research (49, 50). HRV reflects autonomic nervous system balance, with higher HRV typically suggesting better cardiovascular health. The present study investigated the effects of music with different BPM values on HRV and post-exercise recovery to determine whether specific tempo conditions were associated with a more favorable autonomic recovery profile (51). The findings may contribute to the development of non-pharmacological strategies aimed at improving post-exercise autonomic recovery; however, their potential role in reducing SCD risk after physical exertion requires further validation in larger and more diverse populations. These findings may help inform non-pharmacological strategies aimed at improving post-exercise autonomic recovery; however, whether these physiological improvements translate into a reduced incidence of exercise-related arrhythmias or SCD remains speculative and requires confirmation in prospective studies with clinical outcomes. To ensure methodological rigor and comparability among experimental conditions, one-way ANOVA was used, which confirmed the absence of significant intergroup differences in baseline HRV indices or anaerobic cycling performance (all *p* > 0.05), indicating that all groups started from a comparable physiological condition. Group allocation was iteratively adjusted until no significant intergroup differences were observed in these baseline parameters (*p* > 0.05), thus ensuring a uniform physiological starting point.

After high-intensity exercise, the 120 BPM group generally showed a more favorable post-exercise recovery profile, especially in HR reduction and selected HRV indices. However, the results did not demonstrate that 120 BPM was consistently superior across all HRV indicators or recovery time points. Therefore, the present findings should be interpreted as evidence of a partially tempo-dependent recovery response rather than definitive evidence that 120 BPM represents a single optimal recovery tempo recovery.

Importantly, the 130 BPM group demonstrated recovery responses comparable to those observed in the 120 BPM group for several parameters. For example, at 15 min of recovery, the HRV index in the 130 BPM group was numerically higher than that observed in the 120 BPM group, although the overall interaction effect for HRV index was not statistically significant. These findings suggest that the relationship between music tempo and autonomic recovery may not follow a simple linear or single-peak pattern. Instead, different tempos may preferentially influence distinct physiological dimensions of recovery depending on the outcome measure, recovery phase, individual responsiveness, or musical context.

The favorable HR recovery observed in the 120 BPM group may nevertheless have important physiological implications. Rapid HR decline immediately after high-intensity exercise primarily reflects prompt parasympathetic reactivation and the suppression of sympathetic tone (52). In healthy individuals, a sharp decline in HR within the first minute after exercise indicates well-functioning autonomic regulation (53), whereas delayed HR recovery independently predicts cardiovascular morbidity and increased SCD risk (47, 49). Collectively, music at 120 BPM significantly enhances HR recovery by boosting parasympathetic activity and facilitating autonomic rebalancing, which may contribute to reduced post-exercise cardiac vulnerability under the present experimental conditions. Furthermore, the generally higher SDNN values observed during the recovery phase in the 120 BPM group reinforces the efficacy of using this tempo in restoring HRV. SDNN, a key time-domain measure of HRV, reflects the combined effects of both sympathetic and parasympathetic activities. Higher SDNN values are typically associated with stronger autonomic flexibility and cardiac stability (48). A rapid increase in SDNN after exercise suggests a shift toward parasympathetic dominance and the recovery of baseline cardiac function (33). Conversely, delayed or low SDNN recovery may reflect autonomic dysfunction and increased susceptibility to exercise-induced sudden death (33, 48, 49). Hence, monitoring SDNN fluctuations facilitates not only the assessment of individual recovery profiles but also the early identification of high-risk individuals.

Moreover, the 120 BPM group demonstrated better outcomes across HRV metrics during the recovery phase, indicating enhanced autonomic nervous system balance. LF primarily reflects a combination of sympathetic and parasympathetic activities, whereas HF indicates parasympathetic tone. HFnorm represents the relative contribution of parasympathetic activity to overall autonomic activity (47). The HRV index indicates cardiac autonomic function, with higher values denoting better regulatory capacity (50). Impaired cardiac recovery—characterized by low HF and HFnorm or elevated LFnorm—indicates sympathetic overactivation, which may increase the risk of arrhythmias and adverse cardiovascular events (54). Accordingly, these findings suggest that, within the BPM range and experimental conditions examined in this study, 120 BPM music may be associated with more favorable parasympathetic reactivation, reduced relative sympathetic predominance, and improved autonomic re-equilibration.

A possible physiological explanation for the relatively favorable response observed under the 120 BPM condition may involve auditory–cardiorespiratory entrainment and resonance-related autonomic modulation. Previous research has suggested that rhythmic auditory stimulation can influence cardiovascular and respiratory oscillations and that the physiological effects of music tempo may be mediated partly through respiratory synchronization (55, 56). In the present study, 120 BPM corresponds to 2 beats per second, providing a highly regular rhythmic cue during recovery. Although 120 BPM itself is substantially faster than the classical respiratory resonance frequency, repeated rhythmic groupings may theoretically support subharmonic synchronization processes between music rhythm and slower physiological rhythms (57, 58). For example, a 10-s respiratory cycle, corresponding to approximately 6 breaths per minute or 0.1 Hz, could theoretically align with repeated musical beat groupings under a 120 BPM condition (56, 57).

This interpretation is broadly consistent with the concept of cardiovascular resonance. The baroreflex system demonstrates resonance-like behavior near 0.1 Hz, corresponding to approximately six respiratory cycles per minute (57, 58). Breathing near this frequency has been shown to enhance respiratory sinus arrhythmia, increase HRV amplitude, and improve baroreflex sensitivity (57–59). It is therefore plausible that 120 BPM music indirectly facilitated autonomic recovery by stabilizing respiratory rhythm, strengthening cardiorespiratory coupling, or enhancing vagally mediated cardiovascular oscillations during the recovery period (56–60). Such mechanisms may partly explain the greater HR reduction and relatively favorable HRV responses observed in the 120 BPM group.

However, these proposed mechanisms remain hypothetical because the present study did not directly assess respiratory rate, respiratory depth, baroreflex sensitivity, beat-to-beat blood pressure, or phase synchronization between music rhythm and physiological oscillations. Consequently, the present findings should be interpreted as tempo-specific empirical observations under the experimental conditions employed rather than as definitive evidence that 120 BPM directly induces baroreflex resonance or cardiorespiratory synchronization. Future studies should simultaneously measure respiration, blood pressure variability, HRV dynamics, and subjective arousal states while applying time–frequency or phase-locking analyses to clarify whether specific music tempos facilitate autonomic recovery through resonance-related mechanisms (58, 59).

Because respiratory activity was not continuously monitored during recovery, interpretation of HF-related outcomes also warrants caution. HF power is strongly influenced by respiratory sinus arrhythmia, and music tempo may alter respiratory patterns through auditory entrainment effects. Therefore, differences in HF or HFnorm among groups may partly reflect tempo-induced variations in breathing behavior rather than purely altered parasympathetic activity. For this reason, the present interpretation emphasizes the overall pattern of HR recovery together with combined HRV responses rather than relying exclusively on isolated HF-related measures.

However, HRV improvement should be regarded as a surrogate marker of autonomic recovery rather than direct evidence of reduced exercise-induced arrhythmias or SCD incidence.

### Limitations

Several limitations of this study should be acknowledged. First, although the participants were statistically comparable across groups with respect to age, anthropometric characteristics, baseline HRV parameters, and anaerobic cycling performance, the overall sample size remained relatively small. Moreover, the study population primarily consisted of young, healthy, high-performance male athletes. Consequently, the findings cannot be directly generalized to female athletes, recreationally active individuals, older adults, or populations with different physiological or clinical characteristics.

Second, the present study focused primarily on the influence of music tempo, particularly 120 BPM, and did not systematically compare different musical genres, tonal structures, emotional characteristics, or compositional styles, which may restrict the broader applicability of the conclusions.

Third, all BPM conditions were derived from a single instrumental piece, Jay Chou's Blue and White Porcelain, using algorithmic tempo modification. Although this design minimized variability in melody, harmony, timbre, and overall musical structure, it also introduced important interpretative limitations. Because all tempo conditions originated from the same musical composition, the observed physiological responses cannot be attributed exclusively to tempo with complete certainty. Tempo manipulation may have interacted with the original melodic contour, rhythmic phrasing, timbral qualities, and emotional expression of the piece. Moreover, even pitch-preserving tempo adjustment may alter perceived naturalness, groove, emotional valence, and arousal. Participants’ familiarity with and preference for this song were not assessed or statistically controlled, and these factors may have influenced psychological responses and subsequent autonomic regulation. Accordingly, the relatively favorable recovery pattern observed in the 120 BPM condition may reflect the interaction between this specific tempo and the musical characteristics of the selected composition, rather than a universally superior physiological effect of 120 BPM music itself. Furthermore, although pitch-preserving tempo adjustment minimizes alterations in tonal structure, it does not completely preserve perceived musical naturalness, groove, emotional valence, arousal, or listening comfort. Accelerating or slowing a single musical piece may cause listeners to perceive different versions as more or less natural, pleasant, emotionally engaging, familiar, or relaxing. These perceptual differences may independently influence emotional regulation, attentional engagement, and autonomic recovery. Future studies should therefore include multiple musical pieces with different melodic, harmonic, rhythmic, timbral, and emotional characteristics. In addition, future protocols should evaluate familiarity, preference, perceived arousal, emotional valence, musical naturalness, and other music-related psychological variables to clarify whether the observed findings represent a generalized tempo effect or an interaction between tempo and specific musical features.

Fourth, although the favorable recovery response observed in the 120 BPM condition may be partly explained by auditory–cardiorespiratory entrainment, subharmonic synchronization, and resonance-related autonomic regulation, the present study did not directly assess respiratory rhythm, respiratory depth, beat-to-beat blood pressure, baroreflex sensitivity, or phase synchronization between musical rhythm and physiological oscillations. Therefore, the mechanistic relationship between 120 BPM music and enhanced autonomic recovery cannot be definitively established from the current data. The proposed involvement of cardiovascular resonance around 0.1 Hz should therefore be regarded as a plausible theoretical framework rather than direct mechanistic evidence.

Fifth, respiratory control was standardized only during the baseline resting measurement, whereas respiratory rate and tidal volume were not continuously monitored throughout the post-exercise recovery period. This methodological limitation is important because HF HRV and HFnorm are strongly influenced by respiratory sinus arrhythmia, and different BPM conditions may have induced distinct breathing patterns during recovery. As a result, between-group differences in HF-related parameters may have been partially influenced by uncontrolled respiratory variation rather than reflecting parasympathetic modulation alone. Future investigations should incorporate continuous respiratory monitoring and include respiratory variables as covariates in statistical analyses, particularly when interpreting frequency-domain HRV indices during recovery after exercise.

Sixth, HRV measurements were obtained only during three recovery windows: 0–1 min, 5–6 min, and 15–16 min post-exercise. The absence of additional intermediate recovery assessments, particularly around 10 min, limited characterization of the complete temporal trajectory of autonomic recovery. Moreover, the use of ultra-short-term 1-min HRV recordings may reduce the reliability of frequency-domain estimates, including LF, HF, LFnorm, HFnorm, and LF/HF, compared with the conventional 5-min recordings typically recommended for short-term spectral HRV analysis. Accordingly, the frequency-domain findings should be interpreted cautiously and considered exploratory supportive evidence rather than definitive indicators of autonomic modulation. Future studies should incorporate additional recovery time points and employ conventional 5-min HRV recordings whenever feasible.

Finally, the present study evaluated only the acute effects of music intervention during a single recovery session. Therefore, the medium- and long-term sustainability of the observed autonomic benefits remains unknown. In addition, although the experimental environment was standardized as much as possible, external factors such as temperature and humidity may still have influenced HRV measurements and physiological responses. More rigorous environmental control is therefore recommended in future research to improve measurement consistency and data accuracy.

Taken together, further investigations involving larger and more diverse populations, different athletic groups, multiple exercise protocols, various musical stimuli, direct respiratory and cardiovascular measurements, baroreflex sensitivity assessment, phase-synchronization analyses, longer follow-up periods, and stricter control of music-related psychological variables are warranted.

## Conclusion

This study systematically investigated the effects of music tempo, expressed in BPM, on HR and HRV during recovery following high-intensity exercise. Within the experimental conditions and BPM range examined, 120 BPM music was associated with a generally favorable autonomic recovery profile after exercise, primarily reflected by greater HR reduction and relatively beneficial HRV responses, including lower LFnorm and higher HFnorm values during recovery.

However, these findings should be interpreted cautiously and should not be overstated. The 120 BPM condition was not consistently superior across all HRV indices or recovery time points. In particular, the 130 BPM group demonstrated a numerically higher HRV index at 15 min of recovery compared with the 120 BPM group. Therefore, the present findings should not be interpreted as evidence that 120 BPM represents a universally optimal or consistently superior tempo for autonomic recovery. Rather, 120 BPM should be viewed as one tempo condition associated with favorable recovery responses within the specific experimental context of this study, while nearby tempo ranges, including 130 BPM, may also provide beneficial effects for certain HRV outcomes.

One possible explanation is that 120 BPM music may provide a relatively stable auditory rhythmic stimulus that facilitates cardiorespiratory regulation during the early post-exercise recovery period. Through auditory–physiological entrainment and potential subharmonic alignment with slower respiratory and cardiovascular oscillations, this tempo may support parasympathetic reactivation and autonomic re-equilibration. Nevertheless, because respiration, beat-to-beat blood pressure, baroreflex sensitivity, and phase synchronization were not directly measured, these proposed mechanisms remain speculative and require direct experimental confirmation.

Importantly, although improved HRV indices may indicate enhanced autonomic regulation, they do not directly demonstrate a reduction in clinically significant outcomes such as post-exercise arrhythmias or sudden cardiac death risk. Therefore, any potential cardioprotective implication of 120 BPM music should be regarded as hypothesis-generating rather than clinically established. Validation in larger and more diverse populations, with longer monitoring periods and clinically relevant cardiovascular outcomes, is required before broader conclusions can be drawn. Future studies should directly compare 120 BPM and 130 BPM conditions using adequately powered pairwise analyses, multiple musical stimuli, longer recovery monitoring periods, and direct physiological measurements to determine whether one tempo is genuinely superior or whether both tempos belong to a broader favorable tempo range for post-exercise recovery.

Overall, tempo-based music intervention may represent a simple, safe, non-invasive, and non-pharmacological strategy for supporting autonomic recovery after high-intensity exercise under controlled experimental conditions. Nevertheless, broader application of this intervention should be approached cautiously until further mechanistic and clinical evidence becomes available. @@ 60

## Data Availability

The datasets presented in this study can be found in online repositories. The names of the repository/repositories and accession number(s) can be found in the article/Supplementary Material.
